# Kratom Use Within the Context of the Evolving Opioid Crisis and the COVID-19 Pandemic in the United States

**DOI:** 10.3389/fphar.2021.729220

**Published:** 2021-08-26

**Authors:** Walter C. Prozialeck, Peter C. Lamar, Michael Krupp, Matthew Moon, Laura E. Phelps, Oliver Grundmann

**Affiliations:** ^1^ Department of Pharmacology, Midwestern University, Downers Grove, IL, United States; ^2^ Department of Medicinal Chemistry, College of Pharmacy, University of Florida, Gainesville, FL, United States

**Keywords:** kratom, opioid crisis, COVID-19 pandemic, drug abuse, opioid use disorder

## Abstract

Kratom (*Mitragyna speciosa*, Korth.) is an evergreen tree that is indigenous to Southeast Asia. When ingested, kratom leaves or decoctions from the leaves have been reported to produce complex stimulant and opioid-like effects. For generations, native populations in Southeast Asia have used kratom products to stave off fatigue, improve mood, alleviate pain and manage symptoms of opioid withdrawal. Despite the long history of kratom use in Asia, it is only within the past 10–20 years that kratom has emerged as an important herbal agent in the United States, where it is being used for the self-treatment of pain, opioid withdrawal symptoms, and mood disorders. The increase in the use of kratom in the United States has coincided with the serious epidemic of opioid abuse and dependence. Since 2015, efforts to restrict access to prescription opioids have resulted in a marked increase in the use of “street” opioids such as heroin and illicit fentanyl. At the same time, many patients with chronic pain conditions or opioid use disorder have been denied access to appropriate medical help. The lack of access to care for patients with chronic pain and opioid use disorder has been magnified by the emergence of the COVID-19 pandemic. In this report, we highlight how these converging factors have led to a surge in interest in kratom as a potential harm reduction agent in the treatment of pain and opioid use disorder.

## Introduction

Kratom (also known as ketum) is a tree-like plant (*Mitragyna speciosa*, Korth) that is native to Thailand, Malaysia, Indonesia and other regions of Southeast Asia (Adkins et al., 2011; Prozialeck et al., 2012; Cinosi et al., 2015). For generations, indigenous peoples in Southeast Asia have used fresh kratom leaves (either unprocessed or brewed into teas or other decoctions) as a mild stimulant to stave off fatigue, or as an opioid substitute to treat pain or opioid use disorder (Vicknasingam et al., 2010; Singh et al., 2016). Pharmacologic studies have shown that kratom leaves contain over 40 active alkaloids with two of the best characterized being mitragynine and 7-hydroxymitragynine (Adkins et al., 2011; Prozialeck et al., 2012; Kruegel and Grundmann, 2018; Raffa et al., 2018; Prozialeck et al., 2019). Mitragynine has partial biased activity at mu-type opioid receptors, mixed activities at delta opioid receptors, and a variety of effects on other neurotransmitter systems in the central nervous system (Kruegel et al., 2016; Varadi et al., 2016; Obeng et al., 2020; Todd et al., 2020).

Even though kratom has been used in Southeast Asia for generations, it is only over the past 10–20 years that kratom use has expanded to Europe and North America (Prozialeck et al., 2012; Grundmann, 2017). In the United States, kratom products are used extensively for the self-management of pain, opioid use disorder and depression (Swogger et al., 2015; Grundmann, 2017; Swogger and Walsh, 2018; Schimmel et al., 2021). It has been estimated that there may be as many as 1–3 million kratom users in the United States (Prozialeck et al., 2019; Palamar, 2021; Schimmel et al., 2021). The most widely used products include chopped or powdered, dried leaf material (either bulk or in capsule or tablet form) or concentrated extracts that are formulated as teas or capsules (Prozialeck et al., 2012; Grundmann, 2017; Prozialeck et al., 2019; Wilson et al., 2020). These products are widely available from internet vendors or in specialty stores commonly known as “head shops” or “smoke shops”, although some products are now being sold through chain stores that specialize in the sale of herbal supplements.

Kratom is regarded as a new dietary ingredient under the United States Food and Drug Administration (FDA) Dietary Supplement Health and Education Act (for reviews see: (Henningfield et al., 2018; Prozialeck et al., 2019)). Although it remains legal in most of the United States, at the time of writing, several states, such as Alabama, Florida, Indiana, Arkansas, Wisconsin and Tennessee, have passed legislation banning the local sale and possession of kratom (Prozialeck et al., 2019; AKA, 2020). At the same time, several states are in the process of adopting so called “kratom consumer protection acts”, which allow for the sale and use of kratom, but also include standards for the quality control of kratom products (AKA, 2020).

### Evidence for Increased Interest in Kratom

**FIGURE 1:**
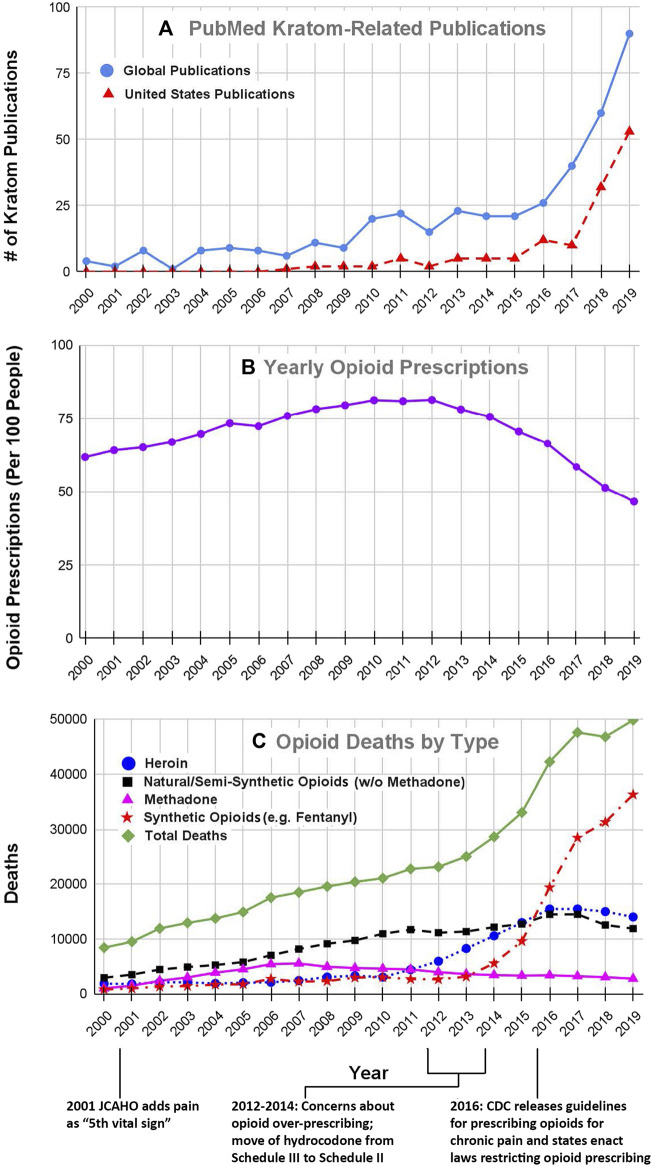
Kratom-related publications **(A)**, opioid prescribing rates **(B)** and number of opioid overdose deaths **(C)** for years 2000–2019. The number of Kratom-related publications (Figure 1A) was obtained from a search of the United States National Library of Medicine’s PubMed database, using the search term “kratom” on april 26, 2021. The solid black line shows the total number of publications for each year, whereas the broken line shows the number of publications in which at least one author was based in the United States. The data for opioid prescribing rates for the years 2000–2006 were obtained from (Kenan et al., 2012); data for the years 2006–2019 were obtained from the CDC database at https://www.cdc.gov/drugoverdose/maps/rxrate-maps.html. Each point represents the number of prescriptions per 100 people for each year. Please note that only 10–20% of people received *any* prescriptions which indicates that people received multiple prescriptions within the same year. Data for the number of opioid-related overdose deaths by drug type were obtained from both the CDC and NIDA data bases at https://www.cdc.gov/nchs/data/databriefs/db394-tables-508.pdf#page=3 and http://www.drugabuse.gov/drug-topics/trends-statistics/overdose-death-rates. All of these databases are in the public domain. The data that were extracted and used for these analyses are included as a supplemental table.

The emergence of kratom as a product or drug of interest in the United States is evident from the results of our literature searches. Our search of the US National Library of Medicine’s PubMed database in April 2021 using the keyword “kratom” yielded a total of 517 articles and reviews. Figure 1A shows the number of kratom articles that were published each year in the period from 2000–2019 which was just before the emergence of the COVID-19 pandemic. As may be seen in the solid blue line in Figure 1, the total number of articles increased steadily from an average of fewer than five per year in the early 2000s to over 90 per year in 2019. Since 2019, this trend has continued, with 91 publications in 2020 and 42 from January-April of 2021 (data not shown in graph). In conjunction with this literature review, we also searched the list of authors for each article to identify papers in which at least some of the work originated in the United States. The broken red line in Figure 1A shows the number of kratom-related articles in which at least one of the authors was based in the United States. We identified a total of 218 articles that were published between 2000 and 2019. Interestingly, we found no papers with American-based authors before 2007. However, since then, the number of articles with American authors has increased markedly. This trend has continued to the present day. In 2020, there were 57 such articles and from January-April of 2021 there were 25 such articles. These results clearly show that the interest in kratom among American researchers has increased markedly over the past decade.

A second line of evidence showing the increased interest in kratom involves the mentions of kratom in reports to poison control centers in the United States. In 2016 Anwar and co-workers analyzed data from the United States National Poison Data System and found that from 2010 to 2015 the number of reports of kratom toxicity increased from about 20 per year to over 250 per year. Subsequent studies showed this trend was also evident for the time period from 2011 to 2018 (Davidson et al., 2021; Eggleston et al., 2019; O'Neill-Dee et al., 2019; Olsen et al., 2019). Anwar et al. (2016) had also reported that as many as 11 deaths in the 2010–2015 time frame may have been at least partly attributable to kratom, although the exact role of kratom in the deaths is unclear (Cumpston et al., 2018; Wing, 2018; Corkery et al., 2019; Hicks, 2019). The issues of kratom toxicity and kratom-related deaths are considered in more detail later.

One additional factor that may have facilitated interest in kratom in the early 2000s was the rapid development of internet communication that became available to increasing numbers of people around the world in the early part of the 21st century (Prozialeck et al., 2012; Williams and Nikitin, 2020). With the increased ease of internet communication, information about kratom, which had been little-known outside of Southeast Asia, could be rapidly disseminated globally.

Various investigators have noted that the increased interest in kratom seemed to coincide with several aspects of the evolving opioid crisis in the United States (Boyer et al., 2008; Prozialeck et al., 2012; Prozialeck, 2016; Bestha, 2018; Coe et al., 2019; Prozialeck et al., 2019) and that recent restrictions on access to prescription opioids for pain management may have further increased demand for kratom (Prozialeck, 2016; Prozialeck et al., 2019). With the emergence of COVID-19 in 2019, and the evolution of the COVID-19 pandemic in 2020, many patients may have faced even further reductions in access to prescription opioids, which could have contributed to an increase in the use of illicit “street” opioids such as heroin, fentanyl and new fentanyl analogs (Manchikanti et al., 2021; Nguyen and Buxton, 2021). It seems likely that this increase in the use of street opioids and the concomitant problems of opioid dependence may have further increased demand for kratom. In this report, we trace the evolution of kratom use in the United states and highlight the likely associations among the development of the ongoing opioid crisis, the unintended consequences of efforts to restrict access to prescription opioids for pain management, and the possible impact of the COVID-19 pandemic on demand for kratom.

### Origins and Evolution of the Ongoing Opioid Crisis in the United States

Opium and drugs derived from its analogs (both natural and synthetic) have been used throughout human history for the management of pain and other conditions such as cough and diarrhea (for review see (Hanson et al., 2006)). In addition, these opioid substances have long been used and abused for their euphoric effects. Attitudes among medical professionals and the American public regarding the use of opioids for pain management have changed and fluctuated over the years (for reviews see, (Ray, 1996; Hanson et al., 2006)).

In the 19th century, opioids were not regulated and were widely available for use without medical guidance (Ray, 1996; Hanson et al., 2006). This led to widespread opioid abuse and dependence that resulted in the passage of federal laws restricting access to opioids. Throughout the first half of the 20th century, the resulting regulations reduced opioid use. However, in the 1960s the use of both medical and recreational opioids soared (Ray, 1996; Hanson et al., 2006). This surge resulted in changes in regulations and attitudes that further restricted access to opioids, a situation that persisted until the mid-1990s and the early 2000s, when the aggressive marketing of opioid products such as Oxycontin® and major changes in regulatory policies resulted in a marked increase in the prescribing of prescription opioids for pain management (Rummans et al., 2018). Figure 1B shows the number of opioid prescriptions/100 people in the United States for each year from 2000 to 2019. Note that the number of opioid prescriptions increased steadily from 2000 to 2012 at which point numbers plateaued and then by 2015 began to decline. We suggest that the increase in opioid prescribing in the early 2000s can be traced to two factors; the policy changes that were instituted by the Joint Commission on Accreditation of Healthcare Organizations (JCAHO) in the year 2000–2001 and the promotion by pharmaceutical companies of opioid products such as Oxycontin® that were touted as safe for use in chronic pain.

In 2000, JCAHO was the primary organization of associations involved in the accreditation of hospitals and other healthcare organizations in the US. A special JCAHO task force had found that many patients throughout the US were suffering from serious pain that was not being treated adequately by their healthcare providers. In response to this situation, JCAHO incorporated specific requirements regarding proper pain assessment and pain management into their accreditation standards, which required that pain be assessed as if it were a “vital sign” (Berry and Dahl, 2000; Phillips, 2000). In cases where the patient reported significant levels of pain, the standards mandated appropriate pain management interventions, which often included various drugs, particularly opioid analgesics. Over the years, JCAHO was reorganized and renamed as the “Joint Commission” and their pain management standards were modified. It is also important to note the initial changes in JCAHO/Joint Commission policies occurred in 2001, a few years after the company Purdue Pharma, had released OxyContin®, a sustained release form of the opioid oxycodone and aggressively marketed the product as safe for treatment of chronic pain (Manchikanti et al., 2012; Thompson et al., 2012; Rummans et al., 2018).

In this environment, physicians and other healthcare providers were under increased pressure to prescribe analgesic drugs (Manchikanti et al., 2012; Rummans et al., 2018), and as may be seen in Figure 1B, the number of prescriptions for opioid analgesics rose markedly from 2000 to 2012.

### Recognition of the Opioid Crisis and Unintended Consequences of Drug Policy Decisions

This increase in opioid prescribing resulted in concomitant increases in opioid abuse and deaths involving both prescription opioids (mainly morphine, codeine, hydrocodone and oxycodone) and “street drugs”, such as heroin and fentanyl. Figure 1C summarizes data for opioid overdose deaths for the period from 2000 to 2019. The rate of overdose deaths from prescription opioids, both natural and semisynthetic (represented by the broken black line in Figure 1B) increased from 2,917 in the year 2000 to over 11,000 in 2011, while deaths from street drugs such as heroin (blue circles in Figure 1B) increased only slightly during the same time period.

By the year 2012, it was readily apparent from the soaring rate of opioid-related overdose deaths that the opioid problem in the US had grown to alarming proportions. This drew the attention of many investigators in the biomedical research community and caused federal regulatory agencies such as the US Centers for disease Control and Prevention (CDC), FDA and US Drug Enforcement Administration (DEA) to begin interventions that were intended to reduce the use of prescription opioids (CDC, 2012; Manchikanti et al., 2012; Nelson & Perrone, 2012; Stayner and Copenhaver, 2012; Rummans et al., 2018). For example, pharmaceutical companies began to develop abuse-deterrent formulations of opioids such as oxycodone (Schaeffer, 2012). In 2014, the DEA moved hydrocodone and products containing it from Schedule III to Schedule II under the Controlled Substance Act (DEA, 2014). Two years later, the CDC followed suit and developed new guidelines to discourage the use of opioids for the management of chronic non-cancer pain (Dowell et al., 2016). Even though the CDC actions were intended to serve as “guidelines”, many clinicians, practice groups, healthcare systems, and even legal authorities, interpreted the “guidelines” as absolute requirements, and some local jurisdictions led by the state of Washington enacted laws to restrict the prescribing of opioids for many types of pain (Franklin et al., 2012; Stolbach and Nelson, 2016; Brookes, 2019). These interventions appeared to achieve the intended goal as evidenced by a marked decrease in the number of opioid prescriptions beginning in 2012 and continuing through 2019 (see Figure 1B).

Unfortunately, these efforts to restrict access to prescription opioids also may have had several unintended consequences, the most notable being a marked increase in the use of street opioids, such as heroin and fentanyl, and an alarming increase in the number of opioid-related overdose deaths. It should be noted that the surge in abuse of street opioids was also driven by a surge in the supply of heroin followed by fentanyl and its analogs being smuggled into the country. The recent surge in deaths from fentanyl and its analogs (broken red line in Figure 1C) is poses a major public health challenge.

### Impact of the COVID-19 Pandemic on Supply and Demand for Kratom

The current COVID-19 pandemic first emerged from China in the late fall to early winter of 2019 and has had a major impact on almost all aspects of healthcare delivery around the world (WHO, 2021). The impact of COVID-19 on supply and demand for kratom has been complex, variable and somewhat unpredictable (Singh et al., 2020). In light of the many case reports and kratom user summaries claiming that kratom has beneficial analgesic and mood-enhancing effects (Prevete et al., 2021), some COVID-19 patients have turned to kratom as a means of treating the pain, lethargy and depression that are commonly associated with COVID-19 infections (Metastasio et al., 2020; Singh et al., 2020). While we are aware of no direct studies showing that kratom can slow transmission and progression of COVID-19 infection, there is one case report suggesting that kratom can alleviate the pain, lethargy and lack of energy that are often experienced by COVID-19 patients (Metastasio et al., 2020). Further studies are needed to clarify this issue.

Besides its direct impact on public health in the US, the COVID-19 pandemic also has had many subtle and less direct effects. For example, many people with non-COVID health issues have encountered difficulties in obtaining appropriate care for their non-COVID problems, as national health leaders called for hospitals to forgo routine visits as well as screening and elective procedures (Puntillo et al., 2020; Caton et al., 2021; Jacka et al., 2021; Kedia et al., 2021; Linas et al., 2021; Mun et al., 2021; Peckham et al., 2021). The lack of access to care has been especially acute for patients with chronic pain problems and/or substance use disorders (Jacka et al., 2021; Kedia et al., 2021; Narayan and Balkrishnan, 2021). The lack of access to medically-assisted care for patients with opioid use disorder during the COVID-19 pandemic has been particularly severe (Jacka et al., 2021; Joudrey et al., 2021). These factors have probably resulted in a well-documented surge in the abuse of street opioids and their many attendant problems (CDC, 2021; Manchikanti et al., 2021). At the same time, it would be expected that the demand for kratom products would increase concomitantly (Singh et al., 2020).

### Problems in Estimating Levels of Kratom Usage in the United States

In preparing this report, we attempted to determine directly how estimates of the levels of kratom usage in the US may have changed over the years. Unfortunately, we were unable to perform such analyses. Data for kratom usage in the late 20th and the early part of the 21st century are not available, and data for use over the past 10 years are quite variable. Results of national survey-based analyses have indicated that there are an estimated 2–3 million kratom users in the United States (Palamar, 2021; Schimmel et al., 2021). However, as Palamar noted (2021), such surveys can often under-estimate usage within the general population. Other estimates of kratom usage based on import data from Indonesia and average kratom consumption may actually be much higher, perhaps as many as 10–20 million (AKA, 2019; Henningfield et al., 2019). Unfortunately, data on levels of kratom imports over the years are not reliable and are skewed by import alerts by the FDA and seizures of kratom shipments in recent years (FDA, 2021). The issue is further complicated by the fact that kratom is currently banned in six states. As a result of these uncertainties, we focused our analyses on better-defined measures of kratom use such as the number of scientific publications and reports of toxicities associated with the use of purported kratom products.

### Kratom Safety Concerns

Over the past decade federal agencies including the CDC, FDA and DEA have raised concerns about kratom toxicity and claimed that there is no evidence that kratom is effective in the treatment of any clinical condition. In 2016, the DEA proposed that kratom’s alkaloid constituents mitragynine and 7-hydroxymitragynine be classified as Schedule I controlled substances, which would have effectively banned the use of kratom in the US (DEA, 2016a; 2016b). The announcement of these plans sparked vigorous opposition from many patients and patient advocacy groups who claim that kratom had helped them manage opioid withdrawal or chronic pain (Anson, 2016; Prozialeck, 2016; Wing, 2016; Prozialeck et al., 2019). The advocates’ responses included a march and demonstration at the White House on September 13, 2016, and a petition was sent to President Obama. In addition, several leading kratom researchers noted that many of the reports of kratom-related deaths may have involved extremely high doses of kratom, the use of kratom products that were adulterated with other drugs, confounding health conditions or the concomitant use of other drugs (Henningfield et al., 2019; Prozialeck et al., 2019; Ramanathan and McCurdy, 2020). In response to these challenges, the director of the DEA announced that the kratom ban would be temporarily placed on hold (DEA, 2016c), a situation that persists to the present day. It should be noted that in February of 2021, it was revealed that the Department of Health and Human Services had actually rescinded the request to move kratom to Schedule I status in 2018, but that information was not released to the public (AKA, 2021).

In evaluating the safety of kratom products it is important to consider kratom within the context of the opioid crisis. In their proposal to schedule kratom, the DEA cited about 44 deaths that may have involved kratom from 2010 to 2016. In that same time frame, over 217,000 people died of opioid poisoning. Overwhelming evidence now indicates that, unlike opioids, kratom does not depress respiratory function to the same degree and is far less dangerous in overdose situations than classic opioids (Henningfield et al., 2019; Prozialeck et al., 2019). In addition, kratom has been shown to reduce craving for opioids in subjects with opioid use disorder (for reviews see (Prozialeck et al., 2019; Sharma and McCurdy, 2021; Singh et al., 2021). In this regard, kratom may have potential as a harm-reduction agent in the treatment of opioid use disorder, similar to cannabis (Ding et al., 2020; Lucas et al., 2021; Socias et al., 2021).

Kratom clearly contains pharmacologically-active compounds and, as such, does have potential for causing toxic effects. Reported toxic effects are actually quite different from those of classic opioids and commonly include: agitation, seizures, arrhythmias and hepatic injury (Eastlack et al., 2020; Kerrigan and Basiliere, 2021; Prozialeck et al., 2012; Schimmel and Dart, 2020). It is important to note, however, that almost all of the reports of toxicity involved the use of kratom products in the West (Davidson et al., 2021; Prozialeck et al., 2019). By contrast, there are few reports of serious adverse effects when kratom products are used in their traditional manner in Southeast Asia (Davidson et al., 2021; Prozialeck et al., 2019; Ramanathan and McCurdy, 2020). This discrepancy suggests that the problem might not be related to the toxicity of kratom per se, but rather the poor quality of some kratom products being sold in the West, including the United States. Various studies indicate that many kratom products may be adulterated with other drugs or be contaminated with toxic metals and infectious microbes (Prozialeck et al., 2019; Prozialeck et al., 2020). In addition to the potential for acute toxicity, kratom can produce a state of physical dependence for which the term “kratom use disorder” has been coined. Dependence on kratom can lead to compulsive use and the appearance of withdrawal symptoms when kratom use is stopped. The symptoms of kratom withdrawal commonly include drug craving, anxiety, insomnia, irritability, and diarrhea (Prozialeck et al., 2012; Singh et al., 2018b). However, the symptoms of kratom withdrawal are quantitatively different, and generally less severe, than those of opioid withdrawal (Singh et al., 2018a; Singh et al., 2021; Stanciu et al., 2021; Vento et al., 2021). Numerous studies have shown that kratom has lower abuse potential than classical opioids (Singh et al., 2018a; Henningfield et al., 2018; Wilson et al., 2021).

### Caveats and Limitations

There are several limitations to the present analyses. As noted previously, hard data on the number of kratom users are not available. As a result, we focused our analyses and discussion on more quantifiable measure of kratom use, such as the number of scientific publications and reports of kratom overdoses. In addition, data on opioid prescriptions and overdose deaths for the year 2020 have not yet been finalized by any federal agencies. To date, the CDC has only issued a report on “Provisional Drug Overdose Death Counts” for the year (CDC, 2021). However, the preliminary data in that report clearly show that the opioid overdose crisis has worsened during 2020, at the same time that the COVID-19 pandemic was evolving. Unfortunately, the data do not necessarily show a cause and effect relationship between the two events. This issue is further complicated by the lack of hard data on the impact of the COVID-19 pandemic on supply and demand for kratom. Based on the available data, we think it is highly likely that the COVID-19 pandemic may have triggered an increase in kratom usage, but additional studies are needed to either confirm or refute this possibility.

### General Perspective and Conclusions

In this discussion, we have postulated that the increased interest in kratom in the US is mainly the result of changing patterns in the use of opioids for pain management and a marked surge in the use of street opioids, such as heroin, fentanyl and emerging fentanyl analogs. In considering this issue, we must also consider the possibility that the increased use of kratom itself may be one of the factors driving the current opioid epidemic. While most researchers are of the opinion that kratom may be useful as a “harm reduction” agent in the treatment of opioid use disorder (McMahon et al., 2019; Prozialeck et al., 2019; Grundmann et al., 2021; Sharma and McCurdy, 2021; Wilson et al., 2021), some have suggested that kratom may be a possible “gateway drug” that can lead users to try harder, more addictive drugs such as street opioids (Tayabali et al., 2018; Schimmel et al., 2021). While this may be the case for a small number of users, it does not seem to be a problem for the vast majority of users (Henningfield et al., 2018; Prozialeck et al., 2019). In fact, there is little or no evidence indicating that kratom is a “gateway” drug for most users (Henningfield et al., 2018; Prozialeck et al., 2019; Garcia-Romeu et al., 2020; Grundmann et al., 2021).

Even though legitimate questions regarding the safety and quality control of kratom products remain to be resolved, the therapeutic potential of kratom and its constituent compounds merit further study. There are numerous active compounds within kratom that appear to have multiple physiologic and psychologic effects beyond analgesia. Online and in-person studies have indicated there may be potential for kratom to produce antidepressant, anxiolytic and antipsychotic effects (Swogger et al., 2015; Grundmann et al., 2018; Coe et al., 2019; Ramanathan and McCurdy, 2020; Grundmann et al., 2021; Sharma and McCurdy, 2021; Smith et al., 2021). In light of these findings, there is clearly a need for further research on safety and efficacy of kratom and its active compounds. With regard to COVID-19, it is well documented that the pandemic has decreased access to medically-assisted treatment for patients with opioid use disorder (Joudrey et al., 2021; Narayan and Balkrishnan, 2021), a situation that leads to an increase in the use of kratom (Prevete et al., 2021). Even though COVID-19 is primarily viewed as a pulmonary disease, infected patients often exhibit symptoms of pain, lethargy and depression. Published analyses of content on kratom user discussion websites indicate that many individuals use kratom to treat such complaints (Smith et al., 2021). Therefore, it seems highly likely that COVID-19 patients may be using kratom for self-medication. There is an urgent need for studies on the impact of the COVID-19 pandemic on levels of kratom usage along with clinical trials of the potential benefits and toxicities of kratom in patients infected with COVID-19.

## Data Availability

The datasets presented in this study can be found in online repositories. The names of the repository/repositories and accession number(s) can be found below: https://www.cdc.gov/drugoverdose/maps/rxrate-maps.html https://www.cdc.gov/nchs/data/databriefs/db394-tables-508. pdf#page = 3 http://www.drugabuse.gov/drug-topics/trends-statistics/overdose-death-rates.
