# Supplementary material for: Kratom Use Within the Context of the Evolving Opioid Crisis and the COVID-19 Pandemic in the United States
Source: Front Pharmacol. 2021 Aug 26;12:729220. doi: 10.3389/fphar.2021.729220 (PMC8427750; doi:10.3389/fphar.2021.729220)
Supplement: Supplementary file 1 [file Table1.DOCX]

Supplementary Table - Raw Data (Prozialeck et al.)

| Year | Opioid Prescriptions (per 100 people) | Heroin Deaths | Prescription Opioid Deaths (Natural/Semi-Synthetic w/o Methadone) | Methadone Deaths | Synthetic Opioids (e.g. Fentanyl) Deaths | Total Opioid Deaths | Global PubMed Kratom Publications | USA PubMed Kratom Publications |
| --- | --- | --- | --- | --- | --- | --- | --- | --- |
| 2000 | 61.9 | 1,842 | 2,917 | 986 | 782 | 8,407 | 4 | 0 |
| 2001 | 64.2 | 1,779 | 3,479 | 1,456 | 957 | 9,496 | 2 | 0 |
| 2002 | 65.2 | 2,089 | 4,416 | 2,358 | 1,295 | 11,920 | 8 | 0 |
| 2003 | 67.0 | 2,080 | 4,867 | 2,972 | 1,400 | 12,940 | 1 | 0 |
| 2004 | 69.8 | 1,878 | 5,231 | 3,845 | 1,664 | 13,756 | 8 | 0 |
| 2005 | 73.4 | 2,009 | 5,774 | 4,460 | 1,742 | 14,918 | 9 | 0 |
| 2006 | 72.4 | 2,088 | 7,017 | 5,406 | 2,707 | 17,545 | 8 | 0 |
| 2007 | 75.9 | 2,399 | 8,158 | 5,518 | 2,213 | 18,516 | 6 | 1 |
| 2008 | 78.2 | 3,041 | 9,119 | 4,924 | 2,306 | 19,582 | 11 | 2 |
| 2009 | 79.5 | 3,278 | 9,735 | 4,696 | 2,946 | 20,422 | 9 | 2 |
| 2010 | 81.2 | 3,036 | 10,943 | 4,577 | 3,007 | 21,089 | 20 | 2 |
| 2011 | 80.9 | 4,397 | 11,693 | 4,418 | 2,666 | 22,784 | 22 | 5 |
| 2012 | 81.3 | 5,925 | 11,140 | 3,932 | 2,628 | 23,166 | 15 | 2 |
| 2013 | 78.1 | 8,257 | 11,346 | 3,591 | 3,105 | 25,052 | 23 | 5 |
| 2014 | 75.6 | 10,574 | 12,159 | 3,400 | 5,544 | 28,647 | 21 | 5 |
| 2015 | 70.6 | 12,989 | 12,727 | 3,301 | 9,580 | 33,091 | 21 | 5 |
| 2016 | 66.5 | 15,469 | 14,487 | 3,373 | 19,413 | 42,249 | 26 | 12 |
| 2017 | 58.5 | 15,482 | 14,495 | 3,194 | 28,466 | 47,600 | 40 | 10 |
| 2018 | 51.4 | 14,996 | 12,552 | 3,023 | 31,335 | 46,802 | 60 | 32 |
| 2019 | 46.7 | 14,019 | 11,886 | 2,740 | 36,359 | 49,860 | 89 | 53 |
| 2020 | - | - | - | - | - | **Estimated**  68,408* | 91 | 57 |

**Data for opioid prescribing rates were obtained from:**

Prescriptions 2000-2006: <https://www.ncbi.nlm.nih.gov/pmc/articles/PMC3659213/>

Prescriptions 2006-2019:<https://www.cdc.gov/drugoverdose/maps/rxrate-maps.html>

-All 3 Accessed 1/7/21

**Data for opioid death statistics were extracted from:**

<https://www.cdc.gov/nchs/data/databriefs/db394-tables-508.pdf#page=3>

-accessed 5/5/2021

*<https://www.commonwealthfund.org/blog/2021/spike-drug-overdose-deaths-during-covid-19-pandemic-and-policy-options-move-forward>

-accessed 4/26/21

**Data for the number of kratom publications were obtained from:**

search of the PubMed database conducted on April 26, 2021.

All of these sources are in the public domain.

*Estimation was made based on total opioid death numbers from January 2020 - August 2020
